# Chemical Composition and Bioactive Characterisation of *Impatiens walleriana*

**DOI:** 10.3390/molecules26051347

**Published:** 2021-03-03

**Authors:** Eleomar de O. Pires, Eliana Pereira, Carla Pereira, Maria Inês Dias, Ricardo C. Calhelha, Ana Ćirić, Marina Soković, Gustavo Hassemer, Carolina Castilho Garcia, Cristina Caleja, Lillian Barros, Isabel C. F. R. Ferreira

**Affiliations:** 1 Centro de Investigação de Montanha (CIMO), Instituto Politécnico de Bragança, Campus de Santa Apolónia, 5300-253 Bragança, Portugal; eleomar.junior@ipb.pt (E.d.O.P.J.); eliana@ipb.pt (E.P.); carlap@ipb.pt (C.P.); maria.ines@ipb.pt (M.I.D.); calhelha@ipb.pt (R.C.C.); ccaleja@ipb.pt (C.C.); iferreira@ipb.pt (I.C.F.R.F.); 2 Departamento Acadêmico de Alimentos (DAALM), Câmpus Medianeira, Universidade Tecnológica Federal do Paraná (UTFPR), CEP, Medianeira, PR 85884-000, Brazil; carolinacgarcia@utfpr.edu.br; 3 Institute for Biological Research “Siniša Stanković”-National Institute of Republic of Serbia, University of Belgrade, 11000 Belgrade, Serbia; rancic@ibiss.bg.ac.rs (A.Ć.); mris@ibiss.bg.ac.rs (M.S.); 4 Câmpus de Três Lagoas, Universidade Federal do Mato Grosso do Sul (UFMS), Três Lagoas, MS 79613-000, Brazil; g.hassemer@ufms.br

**Keywords:** balsaminaceae, bioactivities, food industry, phenolic compounds, promising resource

## Abstract

The attractive colour characteristics of the flowers of the species *Impatiens walleriana* have been arousing great interest in the food industry, which is looking for potential natural sources of colouring ingredients. In this sense, the present work focused on the chemical and bioactive characterization of pink and orange flowers of *I. walleriana*. The phenolic compounds were determined by HPLC-DAD-ESI/MS; in addition, different bioactivities (antioxidant, antimicrobial, anti-inflammatory and cytotoxicity) were also analysed. Both samples studied showed significant amounts of phenolic compounds, especially phenolic acids, flavonoids, and anthocyanins, which justifies the excellent performance in the different bioactivities studied. The orange variety, despite having a greater variety of phenolic compounds, showed a total amount of compounds lower than the pink variety. Overall, the flowers of *I. walleriana* emerge as a promising resource to be explored by the food industry.

## 1. Introduction

The use of flowers in human food is considered an ancestral practice. However, currently, few of these plants are available for daily consumption [1]. Around the world, there are some differences in the consumption habits of edible flowers, which can be justified by the influence exerted by regional customs and culture. There are reports of insertion of edible flowers in various dishes of oriental cuisine, for example, the use of some species (lily, lotus, saffron, rose, and marigold) as ingredients in recipes for cakes and infusions, as well as the application of edible roses in making sauces [2,3]. In Europe, several flowers, such as begonias, marigolds, carnations, chrysanthemums, and clovers, have been used since ancient times in aristocratic banquets for the ornamentation of dishes and drinks [4]. The incorporation of edible flowers in the human diet, in addition to contributing to the concepts of cuisine, makes the dishes more and more attractive, contributes to the addition of aromas, exotic, and delicate flavours, and provides improvements to consumer health, due to the existence of bioactive compounds in their composition, especially phenolic compounds [5,6].

Among the several constituents of flowers, water is the main component of the composition, representing about 70% to 95% of the total flower composition. In addition, the main macronutrients are carbohydrates, followed by proteins and lipids [5]. However, there is some variation in the composition of edible flowers when comparing different studies in the literature, which is generally justified by cultivation conditions (location and environmental and soil conditions) as well as extraction conditions. The literature highlights the presence of phenolic acids and flavonoids (flavones, flavonols, flavanones, and anthocyanins) as predominant bioactive substances in edible flowers and responsible for antioxidant properties [7]. 

Despite not being considered essential nutrients for human vitality, phenolic compounds act as substances with functional properties (antioxidants, antimicrobials, anti-inflammatory and anti-proliferative), which are properties that can also be found in the genus *Impatiens* L. [8]. Popularly known as “Busy lizzy”, *I. walleriana* Hook.f., is an ornamental species, belonging to the family Balsaminaceae A.Rich. [9,10,11]. The species is native to East Africa and popularly recognised for its attractive colourful flowers, which include shades of pink, purple, violet, orange, red, or white [12,13]. Furthermore, the high antioxidant and antifungal activity of the *I. walleriana* extract has auspicious potential and can be used by the food, pharmaceutical, and cosmetic industries [14].

Currently, there is a growing number of studies that characterise and relate the composition of edible flowers with different bioactive properties justified by the growing demand and consumption by the consumer [15].

Thus, this study intends to deepen the knowledge about the phenolic composition of two varieties (pink and orange) of flowers of *I. walleriana*, as well as the evaluation of their bioactivities in order to explore their potential application at an industrial level, namely in the food industry as a natural colourant with functionalising properties.

## 2. Results and Discussion

### 2.1. Colour Evaluation of I. walleriana Flowers

The genus *Impatiens* has attractive flowers in numerous colours, including white, pink, yellow, and orange. Thus, the chromatic analysis in the CIE *L* * *a* * *b* * colour space was carried out on freeze-dried petals of specimens of species *I. walleriana*, in which the proportion according to parameter *L* * was between 0 and 100 and the proportion of parameters *a* * and *b* * was between −120 and 120 (Table 1). The *L* * parameter indicates lightness, so higher values result in clearer objects; the *a* * value indicates the redness–greenness tendency, and the *b* * value indicates the blueness–yellowness tendency.

**Figure 1:**
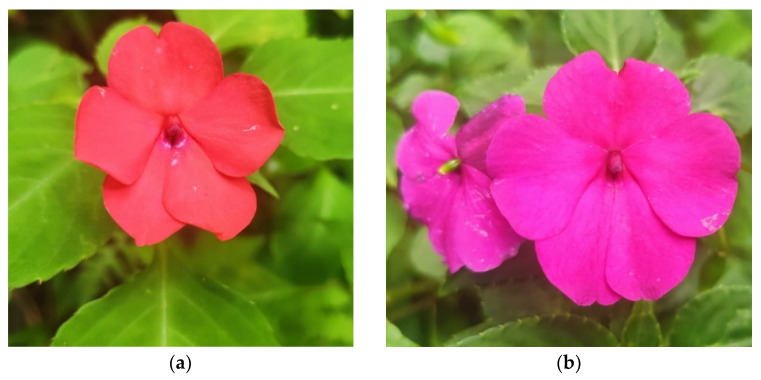
Flower specimens of the *Impatiens walleriana* studied. (**a**): orange petals and (**b**): pink petals (authorship by the author).

Table 1 shows the values obtained in each of the colour parameters *L* *, *a* *, and *b* * for each of the varieties of *I. walleriana* analysed. The corresponding RGB colour is also shown in the same table, which can be verified in the flower (Figure 1). The results show that the orange variety presents values of *L* * and *b* * higher than those presented by the pink variety, translating into a lighter shade and a more yellow range. In turn, the pink variety showed as expected higher values in parameter *a* *, suggesting a more intense shade in the range of reds.

To date, there is no study in the literature on the colourimetry of flowers of *I. walleriana*. However, [16] stated, by chromatography, that the flowers of *I. balsamina* L. may present greater homogeneity of pigments than its stems and sepals. [17] analysed, by spectrophotometry, the pink and orange varieties of flowers of the species *I. walleriana* and *I. hawkeri* W.Bull, which allowed them to state that the anthocyanin content may vary according to the species or part of the plant under study.

### 2.2. Phenolic Composition of I. walleriana Flowers Extracts

The vibrant colours present in flower petals are usually associated with the presence of several compounds found in its composition [18]. The non-anthocyanic and anthocyanic phenolic compounds have been tentatively identified and quantified in both colours of *I. walleriana* and are shown in Table 2. The phenolic profile of chromatrographic peaks referring to the investigated extracts (WO and WP) was characterised by the analysis of the respective parameters, retention time (Rt), maximum absorption wavelength in the UV-Vis region (λ max), deprotonated ion ([M-H]^-^), and molecular ion fragmentation (MSn). Then, attempts were also made to identify them through data previously reported in the literature. In the Supplementary Materials file, we present the chromatograms of both samples for non-anthocyanin and anthocyanin compounds as well as the TIC of the major peaks found. 

With regard to non-anthocyanic compounds, four phenolic acids (derived from caffeic and *p*-coumaric acid) and one flavanone (*O*-glycosylated derivative of eriodictyol) were tentatively identified. To the authors’ knowledge, there is no information regarding the chromatographic profile of the non-anthocyanin phenolic compounds present in both colours of *I. walleriana*. From a more comprehensive perspective, these compounds have been described in agreement with previous descriptions in the literature for other herbaceous genera plant specimens. As a result, peaks 1 ([M − H]^−^ at *m/z* 341) and 2/3/4 ([M − H]^−^ at *m/z* 325) were tentatively identified as caffeic acid and *p*-coumaric acid hexoside, respectively, as described previously in extracts of *Taraxacum sect. Ruderalia* [19] and *Solanum lycopersicum* L. [20]. Peak 5 ([M − H]^−^ at *m*/*z* 449, tentatively identified as eriodictyol-*O*-hexoside, presented a UV-vis spectrum characteristic at λmax 310 nm, having been previously observed by [21] in samples of *Rosa canina* L. Peak 3, *p*-coumaric acid hexoside, was the major compound found in both extracts, presenting concentrations that ranged from 9.21 ± 0.01 mg/g extract in WP samples to 2.92 ± 0.08 mg/g extract in WO samples. Moreover, WP extract presented higher concentrations in total phenolic acids, total flavanones, and total non-anthocyanin phenolic compounds, 15.9 ± 0.3, 2.57 ± 0.01, and 18.6 ± 0.3 mg/g extract, respectively.

Regarding the anthocyanin phenolic compounds, ten anthocyanin phenolic compounds were tentatively identified in the samples of *I. walleriana*: four pelargonidin glycosylated derivatives, three malvidin derivatives, and three peonidine derivatives. Peaks 7/8–10–12/14/15 were linked to phenolic acids residues. The anthocyanin glycosil acylation increases the molecular size and changes the spatial structure of the anthocyanin aglycone, and it usually occurs with low polarity organic acids. This phenomenon decreases the polarity of the molecule and, consequently, increases their retention time in a reversed-phase column [22].

Pelargonidine derivatives are undoubtedly those with the highest numerical expression in the samples. The attempt to identify peaks 6 and 9 ([M + H]^+^ at *m*/*z* 741) as pelargonidin-*O*-hexoside-*O*-deoxyhexoside-hexoside was carried out with the previously described by [23]. In order to identify peaks 11 and 13 ([M + H]^+^ at *m*/*z* 783), such as pelargonidin-*O*-*p*-coumaroyl-hexoside-*O*-acetyl-hexoside, those previously described by [24] was used. Regarding malvidin derivatives, peaks 8 and 10 ([M + H]^+^ at *m*/*z* 801), tentatively identified as malvidin-3-*O*-coumaroylhexoside-*O*-hexoside, have already been properly characterised and identified by other authors [25]. For peak 15, the tentative identification was performed only by the chromatographic data obtained, in which it presented a protonated molecule at [M + H]^+^ at *m*/*z* 843, with subsequent losses of fragments MS^2^ at *m*/*z* 639 (loss of an acetyl residue and an hexose) and *m*/*z* 331 (loss of a residue of *p*-coumaric acid and an hexose), so it has been tentatively identified as malvidin-*O*-acetylhexoside-*O*-coumaroylhexoside. Finally, for peonidine derivatives, no bibliographical references were found to support the tentative identification of these peaks. Peak 7 showed a protonated molecule at [M + H]^+^ at *m*/*z* 771, with an MS^2^ fragment at *m*/*z* 609, corresponding to the loss of an hexose and another MS^2^ fragment at *m*/*z* 301, corresponding to the peonidine aglycone but also to the loss of a *p*-coumaric acid residue and an hexose residue, having therefore been tentatively identified as peonidine-*O*-hexoside-*O*-*p*-coumaroylhexoside. Peaks 12 and 14 were tentatively identified as peonidine-*O*-acetylhexoside-*O*-*p*-coumaroylhexoside, presenting a protonated molecule at [M + H]^+^ at *m*/*z* 813, with subsequent losses of fragments MS^2^ at *m*/*z* 609 (162 u + 42 u, acetyl and hexose residues) and *m*/*z* 301 (146 u + 162 u, *p*-coumaric acid residues and hexose), peonidine aglycone. Although WO extract demonstrated a greater variety of anthocyanin compounds, it was the WP extract that stood out in the higher concentrations of anthocyanins (17.4 ± 1.1 mg/g extract), which was mainly due to malvidin-3-*O*-coumaroylhexoside-*O*-hexoside concentrations (11.5 ± 0.8 mg/g extract). 

Some authors [26] researched the phenolic content of 51 edible and wild flowers. Among its findings, it was observed that the protocatechuic acid (183.45 mg/100 g), epicatechin (96.11 mg/100 g), and the protocatechuic acid (83.97 mg/100 g) were the three main phenolics present in specimens of the species *I. walleriana*. In contrast, the extracts of *I. walleriana* (WO and WP) analysed in our study showed the highest proportions for *p*-coumaric acid, caffeic acid, and eryodictiol-*O*-hexoside. 

[27] studied polyphenols in six species of the genus *Impatiens* (*I. balfourii* Hook.f.; *I. balsamina*; *I. walleriana*; *I. glandulifera* Royle; *I. noli-tangere* L.; *I. parviflora* DC.). Through the phenolic profile corespondent to *I. walleriana*, amounts of total phenolics (12.24 ± 0.08 mg/g dry weight, DW), phenolic acids (2.69 ± 0.08 mg/g DW), flavonoids (3.94 ± 0.29 mg/g DW) were observed. Differences can be justified due to the time of harvest of plants, the organs studied, and climatic conditions, among other external factors [17,28].

[29] reported the presence of malvidin and peonidine derived in samples of *I. noli-tangere*. [30] observed malvidin glucosides in flowers of the species *I. textorii* Miq., while [31] identified derivatives of pelargonidine, peonidine, and malvidin in petals of *I. balsamina*. However, more specifically, [17] studied the anthocyanin content present in the organs (nodules, internal, petioles of leaves, leaves, petals, and pedicels) of the species *I. walleriana* and *I. hawkeri*. The investigation showed that the rose and orange petals of *I. walleriana* presented notable values of anthocyanin compounds that varied among themselves. A factor that was also observed in this study was that the anthocyanin composition of the rose petals was different from the composition of the orange petals, which is in accordance with the major anthocyanin compounds found in both samples. Meaning, the orange samples presented highest concentrations in pelargonidin derivatives, followed by peonidin derivatives, explaining its orange colour. On the other hand, the pink samples presented the highest amounts of malvidin derivatives followed by pelargonidin derivatives, explaining their pink colour. In both cases, the correlation between colours and anthocyanin compounds followed the information previously reported by Tanaka, Sasaki, and Ohmiya [32]. In this particular plant, we do not have enough data to correlate the colour of the studied samples with the presence of acylated anthocyanins; further studies needed to be conducted for that purpose. However, what it is possible to state, being previously described by other authors, is that the acylation of the anthocyanin molecule increases its colour and chemical stability [22,33], making *I. walleriana* flowers extracts a very promising matrix for the food industry, which precisely needs stable natural colouring solutions for the replacement of the artificial ones.

### 2.3. Bioactive Properties of I. walleriana Flowers Extracts

The antioxidant activity of WO and WP extracts was evaluated by the oxidative hemolysis inhibition method (OxHLIA), and the results were expressed in terms of EC_50_, as shown in Table 3. In general, the two extracts analysed showed excellent antioxidant activity. Even so, and considering that the lower the EC_50_ value, the greater the antioxidant capacity, it was possible to verify that the pink petals from *I. walleriana* (34 ± 2 µg/mL, WP) performed better when compared to the orange ones (59 ± 7 µg/mL, WO).

Reference[34] investigated the antioxidant activity in vitro of the aqueous ethanolic extract (80:20, EtOH:H_2_O, *v*/*v*) of whole plants of the species *I. balsamina*, *I. hawkeri*, and *I. walleriana*, highlighting its excellent performance with EC_50_ values of 0.41, 0.10, and 0.44 mg/mL, respectively. In turn, [35] stated that the antioxidant activity of the genus *Impatiens* varies according to the part of the plant and also with the extraction methodology adopted. Such questioning may justify the differences between the results obtained in this work when compared with other studies in the literature. [14] compared three extracts (methanol, hexane, and chloroform) of *I. walleriana*. The results obtained indicated that methanol (80.4%) was the solvent with the best percentage of radical elimination, followed by hexane (70.6%) and chloroform (52.5%), pointing out that the solvent used can interfere in the diagnosis of the antioxidant capacity. Additionally, [35] presents the amount of phenolic compounds as a direct influence on the antioxidant capacity, which, in the present study, appears as correlable.

The effect of both extracts (WO and WP) of *I. walleriana* on the growth of the four human tumour cell lines was determined, and the GI_50_ values (concentrations that caused 50% of the cell growth inhibition) are detailed in Table 3. The results showed that both colours of petals (WO and WR) presented positive results for the three tested cell lines (GI_50_ < 400 μg/mL), being able to inhibit the growth of MCF-7 (breast adenocarcinoma), HeLa (cervical carcinoma), and HepG2 (hepatocellular carcinoma) cell lines in a moderate way. Regarding the NCI-H460 cell line (non-small cell lung cancer), only the orange samples (WO) appears with inhibition ability. None of the extracts demonstrate toxicity against PLP2 cell lines (GI_50_ > 400 μg/mL). The results also revealed that the WO extract showed the best antitumour performance through all the cell lines tested compared to the WP extract. 

[36] evaluated the antioxidant activity and the cytotoxic effect of the aqueous extract of the species *Ocimum basilicum* L. and *I. walleriana* in AGS cell lines of gastric cancer. From their data, it can be seen that the maximum toxicity of the aqueous extract of *I. walleriana* occurred 72 h after adding the extract to the cells, in which a minimum IC_50_ value of 2.87 ± 0.001 mg/mL was found for the AGS line cell. [37] developed a cytotoxic assay from the extract (CHCl_3_ and MeOH) of the plant material of *I. parviflora*, against prostate cancer cell lines (DU145 and PC3) and melanoma (A375, WM793, and HTB140). It was observed that the substance did not produce mutagenic potential for all cell lines tested, and that the best cytotoxicity results were for the A375 cell line. Therefore, it can be observed that the anti-tumour activity found in the present research was similar to the results obtained in the literature, since the results did not present toxicity levels for non-tumour cells and revealed interesting values against several human tumour cell lines.

Regarding the anti-inflammatory activity, both analysed extracts (WO and WP) showed satisfactory results (Table 3) with WO standing out with a lower GI_50_ value (312.1 ± 5.5 µg/mL). No studies were found in the literature regarding the anti-inflammatory activity of *I. walleriana*. However, [29] evaluated the anti-inflammatory activity of the ethanolic extract (50:50, *v*/*v*) of dry leaves and stems of the *I. noli-tangere* species against the inflammatory enzymes lipoxygenase (LOX) and cyclooxygenase (COX-1 and COX-2). The authors stated that the bioactive extracts found are promising sources for the treatment of inflammatory diseases [29].

The difference regarding the anti-inflammatory potential presented by the literature and the values found in this research can be justified by the differences such as parts of the plants studied, extraction method, the proportion of solvent, and because different species were evaluated.

The antimicrobial and antifungal activity of the *Impatiens* extracts was examined against a panel of six species of bacteria (*Bacillus cereus*; *Escherichia coli*; *Listeria monocytogenes*; *Pseudomonas aeruginosa*; *Salmonella typhimurium*; *Staphylococcus aureus*) and six species of fungi (*Aspergillus fumigatus*; *A. niger*; *A. versicolor*; *Penicillium funiculosum*; *P. ochrachloron*; *P. verrucosum* var. *cyclopium*) selected on the basis of their relevance to public health. The data were expressed as minimum inhibitory concentration (MIC), minimum bactericidal concentration (MBC), and minimum fungicidal concentration (MFC), and these are reported in Table 4.

It can be noted that the MIC and MBC values were notable for all bacterial cultures analysed. In addition, *B. cereus* and *E. coli* were the most sensitive bacteria for all extracts due to their lower MIC values of 0.05 mg/mL (WP) for *B. cereus* and 0.075 mg/mL (WO and WP) for *E. coli*. Regarding bactericidal performance (MBC), it was found that *E. coli* was the most susceptible strain, presenting a CMB of 0.10 mg/mL for the two extracts analysed. [27] found the lowest values of minimum inhibitory concentration (0.500 mg/mL, MIC) for strains of *Bacillus subtilis*, *Streptococcus pneumoniae,* and *Streptococcus pyogenes* for *I. walleriana*. In a study developed by [14], it was found that the methanolic extract of *I. walleriana* showed greater antibacterial activity against *E. coli.* [34] observed minimum inhibitory concentration (MIC) values between 5 and >10 mg/mL for a panel of eight different microorganisms (*Aspergillus niger*, *Candida albicans*, *E. coli*, *Pseudomonas aeruginosa*, *Staphylococcus aureus*, *S. epidermidis*, *Streptococcus pneumoniae*, and *S. pyogenes*) for the ethanolic extract of the whole plants of *I. walleriana*. The values found in the literature are in agreement with those obtained in the present study, in order to reinforce the antibacterial potential of the extracts of the flowers of *I. walleriana*.

The results of the antifungal activity of both extracts (WO and WP) showed notoriety for all fungi in the panel, so that the MIC values ranged between 0.025 and 0.05 (mg/mL) and MFC 0.05 to 0.10 (mg/mL). However, no strain was more sensitive than the others, and none of the extracts (WO and WR) performed better, indicating that both extracts play an important role in fighting fungi. Studies of antifungal activity for *I. walleriana* are scarce in the literature, but [14] stated that due to the high antibacterial and antifungal activity of the extract, it has an auspicious potential as a natural antioxidant in the food, pharmaceutical, and cosmetic industry.

## 3. Materials and Methods

### 3.1. Preparation of the Samples

Specimens of *Impatiens walleriana* with orange and pink flowers (Figure 1) were collected in July and August 2019 from a rural property in the municipality of Matelândia (25°15′07.8″ S 53°59′16.1″ W), in the state of Paraná, southern Brazil. Vouchers (*Herbarium specimens*) were deposited in the FLOR herbarium (barcodes: FLOR0067269, FLOR0067270). Immediately after harvesting, the flowers were washed three times in water and dried with absorbent paper. Its petals were removed, stored, and separated according to their colour in plastic containers. The samples were frozen, freeze dried (Freezone 4.5, Labconco, Kandas City, MO, USA), crushed, and stored in airtight bottles protected from light.

### 3.2. Colour Evaluation

The colour of the petals was measured in a colourimeter (model CR-400, Konica Minolta Sensing, Inc., Osaka, Japan) following the methodology used by [38]. The CIE *L* * *a* * *b* * colour space values were recorded using the illuminant C and diaphragm aperture of 8 mm, and the data obtained were processed according to the software “Spectra Magic Nx” (version CM-S100W 2.03. 0006), by Konica Minolta.

### 3.3. Phenolic Composition

#### 3.3.1. Extracts Preparation

The freeze-dried samples (0.5 g) from both colours of *I. walleriana* were submitted to maceration at room temperature with the addition of 30 mL of an ethanol/water solution (80:20 *v*/*v*, for the anthocyanin extraction 0.5% TFA was added to the extracting solvent), during 1 h (150 rpm). Then, the extracts were filtered with a filter paper (Whatman No. 4), and the retained residue was re-extracted through the same procedure. The ethanolic fraction of the extracts were removed under reduced pressure on a rotary evaporator (Büchi R-210, Flawil, Switzerland). Finally, the aqueous phase of all extracts was frozen and freeze dried.

#### 3.3.2. Identification and Quantification of Phenolic Compounds

The freeze-dried extracts were re-dissolved in a ethanol:water (20:80, *v*/*v*) solution and filtrated for a 2 mL HPLC vial for further phenolic compounds analysis. 

The chromatographic analysis for phenolic compounds (non-anthocyanin and anthocyanin compounds) was achieved using a Dionex Ultimate 3000 UPLC system (Thermo Scientific, San Jose, CA, USA) coupled to a DAD detector and mass spectrometer Linear Ion Trap LTQ XL (ThermoFinnigan, San Jose, CA, USA), equipped with an ESI source (electrospray ionisation source), working in negative mode for non-anthocyanin compounds and positive mode for anthocyanin compounds. For non-anthocyanin phenolic compouds analysis, a Waters Spherisorb S3 ODS-2 reverse phase C18 column (4.6 × 150 mm, 3 μm; Waters, Milford, MA, USA) and an elution gradient using as mobile phase formic acid/water (0.1%) and acetonitrile recorded at 280, 330, and 370 nm as preferred wavelengths, as previously described by [39]. For anthocyanin phenolic compounds, a reverse phase AQUA^®^ C18 column (5 µm, 150 mm × 4.6 mm i.d., Phenomenex, Torrance, CA, USA) and an elution gradient using as mobile phase 0,1% TFA in water, and 100% acetonitrile were used, and recorded at 520 nm as the preferred wavelength, as previously described by [40]. For the identification of the compounds, the data obtained (retention times, UV-Vis spectra, and mass spectra) were compared with data available in the literature and, when available, with the standards. For quantitative analysis, 7-level calibration curves were obtained by injection of standard solutions with known concentrations: caffeic acid (*y* = 388,345*x* + 406,369, *R^2^* = 0.9939, LOD (Limit of Detection) = 0.78 µg/mL and LOQ (Limit of Quantification) = 1.97 µg/mL), *p*-coumaric acid (*y* = 301,950*x* + 6966.7, *R^2^* = 1, LOD = 0.68 µg/mL and LOQ = 1.61 µg/mL), naringenin (*y* = 18,433*x* + 78,903, *R^2^* = 0.9994, LOD = 0.17 µg/mL and LOQ = 0.81 µg/mL), and pelargonidin-3-*O*-glucoside (*y* = 268,748 × 71,423; *R²* = 0.9986, LOD = 0.24 µg/mL and LOQ = 0.76 µg/mL). In cases where no standard compound was available, the quantification was performed using the calibration curve of a compound within the same phenolic group. Results were expressed in mg per g of dry extract.

### 3.4. Bioactivities Evaluation

#### 3.4.1. Evaluation of Antioxidant Activity

For antioxidant activity evaluation, both dry extracts were re-dissolved in a PBS solution and diluted successively to allow determining the corresponding EC_50_ values. The oxidative hemolysis inhibition test (OxHLIA) was performed on sheep blood samples, as previously reported by [41]. The results were presented according to the concentration inhibition parameters (EC_50_ value, µg/mL) with the capacity to produce a Δt hemolysis delay in a time of 60 min. Trolox was used as a positive control.

#### 3.4.2. Evaluation of the Cytotoxic and Hepatotoxic Activity

Both *I. walleriana* extracts were tested in four different human tumour cell lines, namely, MCF-7 (breast adenocarcinoma), NCI-H460 (non-small cell lung cancer), HeLa (cervical carcinoma), and HepG2 (hepatocellular carcinoma). The sulforodamine B assay was used to measure the cell growth inhibition following the procedure described by [42]. The hepatotoxic was measured by using a culture of liver cells from freshly harvested pigs (acquired in certified slaughterhouses), called PLP2. 

In both assays, a phase contrast microscope was used to monitor the growth of cell cultures, which were sub-cultured and plated in 96-well plates (density of 1.0 × 10^4^ cells/well). Dulbecco’s modified Eagle’s medium (DMEM) was supplemented with FBS (10%), penicillin (100 U/mL), and streptomycin (100 μg/mL). Ellipticin was used as a positive control, and all the results were expressed as GI_50_ values (μg/mL).

#### 3.4.3. Evaluation of Anti-Inflammatory Activity

The LPS-induced NO production by murine macrophage (RAW 264.7) cell lines was determined as the nitrite concentration in the culture medium [43]. Dexamethasone (50 μM) was used as a positive control, and the results were expressed as IC_50_ values (μg/mL).

#### 3.4.4. Evaluation of Antimicrobial Activity

Antibacterial activity was evaluated according to procedures previously described by [19] using three Gram-positive bacteria *Bacillus cereus* (human isolate), *Staphylococcus aureus* (ATCC 11632), and *Listeria monicytogenes* (NCTC 7973) and three Gram-negative bacteria *Escherichia coli* (ATCC 35210), *Pseudomonas aeruginosa* (ATCC 27853), and *Salmonella typhimurium* (ATCC 13311). The minimum inhibitory concentration (MIC) and minimum bactericidal (MBC) concentration were determined, and streptomycin and ampicillin were used as positive controls. On the other hand, the antifungal activity was evaluated following the protocol described by [44], using *Aspergillus fumigatus* (ATCC 1022), *Aspergillus versicolor* (ATCC 11730), *Aspergillus niger* (ATCC 6275), *Penicillium funiculosum* (ATCC 36839), *Penicillium ochrachloron* (ATCC 9112), and *Penicillium verrucosum* var. cyclopium (food isolate). The MIC and minimum fungicidal concentration (MFC) were determined. Ketokonazole and bifonazole were used as positive controls. The microorganisms were purchased at the Mycology Laboratory of the Department of Plant Physiology of the Institute for Biological Research “Siniša Stanković” at the University of Belgrade, Serbia.

### 3.5. Statistical Analysis

The results are presented as mean ± standard. Student’s *t*-test was applied to assess significant differences between samples, with α = 0.05. Throughout the work, the level of significance is 0.05.

## 4. Conclusions

Although there are some studies on the bioactive components of the genus *Impatiens*, little is known about its phenolic profile, especially with regard to the colouring material present in its flowers. This factor must be taken into account, since its petals have an attractive colour and, consequently, can be a promising source of bioactive compounds.

The present work confirmed that the petals of the flowers of the species *I. walleriana* have a significant phenolic amount, mainly in terms of phenolic acids, flavonoids, and anthocyanins. In general, it is also worth mentioning that the biological activity found (WO and WP) was of great value, since both showed notoriety in performance, especially with regard to the antibacterial, antioxidant, and antifungal activities. As for the colouring aspects, it is noted that the pink petals extract showed the greater intensity of colour and quantity in its phenolic profile, mainly related to the composition of anthocyanins, while orange extract was responsible for a greater variety of compounds.

However, future research is necessary to deeply investigate the knowledge about the flowers of *I. walleriana*, since these can be promising sources of natural raw material for the development of nutraceutical ingredients, such as dyes, antioxidants, and antimicrobial agents for the food industry.

## Figures and Tables

**Table 1:** Colour parameters (CIE *L* * *a* * *b* *) of fresh petals from *Impatiens walleriana* samples (orange colour_WO and pink colour_WP) (mean ± SD).

Colour Parameters	Fresh Petals		
	WO	WP	*p* Value
*L* *	47.9 ± 3.5	36.6 ± 0.9	<0.001
*a* *	39.2 ± 2.2	43.7 ± 1.6	<0.001
*b* *	48.9 ± 3.1	10.7 ± 0.7	<0.001
RGB colour			<0.001

*L* * luminosity; *a* * chromatic axis from green (−) to red (+); *b* *, chromatic axis from blue (−) to yellow (+).

**Table 2:** Retention time (Rt), maximum wavelengths of absorption in the UV-Vis region (λ max), tentative identification, and quantification of the non-anthocyanin and anthocyanin phenolic compounds present in the petal extracts of *Impatiens walleriana* (orange colour_WO and pink colour_WP) (mean ± SD).

Non-Anthocyanin Phenolic Compounds								
Peak	Rt (min)	λ_max_ (nm)	[M − H]^−^	Ms^2^ (*m*/*z*)	Tentative Identification	Quantification (mg/g)		t-Students Test *p*-Value
						WO	WP	
1	5.15	325	341	179 (100), 135 (7)	Caffeic acid hexoside	0.52 ± 0.01	1.74 ± 0.08	<0.01
2	7.11	315	325	163 (100), 145 (71), 119 (11)	*p*-Coumaric acid hexoside	2.92 ± 0.08	5.0 ± 0.2	<0.01
3	7.29	315	325	163 (100), 145 (73), 119 (15)	*p*-Coumaric acid hexoside	2.688 ± 0.004	9.21 ± 0.01	<0.01
4	7.92	311	325	163 (40), 145 (51), 119 (5)	*p*-Coumaric acid hexoside	1.09 ± 0.03	n.d.	<0.01
5	9.21	310	449	287 (49), 269 (100)	Eryodictiol-*O*-hexoside	1.088 ± 0.003	2.57 ± 0.01	<0.01
					TPA	7.22 ± 0.09	15.9 ± 0.3	<0.01
					TFlav	1.088 ± 0.003	2.57 ± 0.01	<0.01
					TNAPC	8.3 ± 0.1	18.6 ± 0.3	<0.01
**Anthocyanin Phenolic Compounds**								
**Peak**	**Rt (min)**	**λ** **_max_** **(nm)**	**[M + H]^+^**	**Main Fragment** **ESI-MSn [Intensity (%)]**	**Tentative Identification**	**Quantification (mg/g)**		**t-Students Test** ***p*- ** **Value**
						WO	WP	
6	34.04	506	741	579 (100), 271 (12)	Pelargonidin-*O*-hexoside-*O*-deoxyhexoside-hexoside	1.04 ± 0.04	n.d.	<0.01
7	35.83	511	771	609 (100), 301 (10)	Peonidin-*O*-hexoside-*O*-*p*-coumaroylhexoside	0.72 ± 0.03	n.d.	<0.01
8	36.62	510	801	639 (25), 331 (100)	Malvidin-3-*O*-*p*-coumaroylhexoside-*O*-hexoside	n.d.	1.43 ± 0.01	<0.01
9	38.48	504	741	579 (100), 271 (15)	Pelargonidin-*O*-hexoside-*O*-deoxyhexoside-hexoside	2.7 ± 0.2	n.d.	<0.01
10	39.73	510	801	331 (100)	Malvidin-3-*O*-*p*-coumaroylhexoside-*O*-hexoside	n.d.	11.5 ± 0.8	<0.01
11	40.28	509	783	579 (100), 475 (34), 271 (25)	Pelargonidin-*O*-*p*-coumaroyl-hexoside-*O*-acetyl-hexoside	1.3 ± 0.1	n.d.	<0.01
12	41.11	511	813	609 (100), 301 (14)	Peonidin-*O*-acetylhexoside-*O*-*p*-coumaroylhexoside	0.8 ± 0.2	n.d.	<0.01
13	41.71	504	783	579 (100), 475 (34), 271 (35)	Pelargonidin-*O*-*p*-coumaroyl-hexoside-*O*-acetyl-hexoside	4.39 ± 0.17	1.2 ± 0.1	<0.01
14	42.48	511	813	609 (100), 301 (12)	Peonidin-*O*-acetylhexoside-*O*-*p*-coumaroylhexoside	3.5 ± 0.2	n.d.	<0.01
15	42.7	511	843	639 (100), 331 (34)	Malvidin-*O*-acetylhexoside-*O*-*p*-coumaroylhexoside	n.d.	3.4 ± 0.3	<0.01
					TAPC	14.4 ± 0.8	17.4 ± 1.1	<0.01

TPA—Total Phenolic Acids; Tflav—Total Flavonoids; TNAPC—Total non-anthocyanin phenolic compounds; TAPC—Total Anthocyanin Phenolic Compounds. n.d.—not detected (below detection limit). Standard calibration curves: caffeic acid (*y* = 388345*x* + 406369, *R^2^* = 0.9939, LOD (Limit of Detection) = 0.78 µg/mL and LOQ (Limit of Quantification) = 1.97 µg/mL, peak 1), *p*-coumaric acid (*y* = 301950*x* + 6966.7, *R^2^* = 1, LOD = 0.68 µg/mL and LOQ = 1.61 µg/mL, peaks 2, 3, and 4), naringenin (*y* = 18433*x* + 78903, *R^2^* = 0.9994, LOD = 0.17 µg/mL and LOQ = 0.81 µg/mL, peak 5), pelargonidin-3-*O*-glucoside (*y* = 268748*x* − 71,423; *R²* = 0.9986, LOD = 0.24 µg/mL and LOQ = 0.76 µg/mL, peaks 6 to 15).

**Table 3:** Results of oxidative hemolysis inhibition assay (OxHLIA), cytotoxic, hepatotoxic, and anti-inflammatory activity of the extracts from *Impatiens walleriana* (orange colour_WO and pink colour_WP) (mean ± SD).

	WO	WP	t-Students Test *p*-Value
**Antioxidant activity (Ec_50_ values; µg/mL)**			
Oxidative hemolysis inhibition assay (OxHLIA)	59 ± 7	34 ± 2	<0.01
**Non-tumour cell lines (GI_50_ values; µg/mL)**			
PLP2	>400	>400	<0.01
**Tumour cell lines (GI_50_ values; µg/mL)**			
HeLa (cervical carcinoma)	177.3 ± 9.9	215.6 ± 8.8	<0.01
HepG2 (hepatocellular carcinoma)	207.1 ± 16.6	277.9 ± 9.2	<0.01
MCF7 (breast adenocarcinoma)	290.9 ± 9.4	307.72 ± 14.9	<0.01
NCI-H460 (non-small cell lung cancer)	333.4 ± 10.5	>400	<0.01
**Anti-inflammatory (GI_50_ values; µg/mL)**			
Inhibition of NO production in LPS stimulated RAW 264.7 cells	312.1 ± 5.5	349.21 ± 12.8	<0.01

EC_50_ values: Extract concentration corresponding to 50% of the antioxidant activity. GI_50_ values: concentration that inhibits 50% of cell growth. Ellipticin GI_50_ values (positive control): 1.21 μg/mL (MCF-7), 1.03 μg/mL (NCI-H460), 0.91 μg/mL (HeLa), 1.10 μg/mL (HepG2), and 2.29 μg/mL (PLP2). GI_50_ > 400 μg/mL has no activity.

**Table 4:** Antibacterial (minimum inhibitory concentration (MIC) and minimum bactericidal concentration (MBC) mg/mL) and antifungal (MIC and MFC mg/mL) activity of *Impatiens walleriana* extracts (orange colour_WO and pink colour_WP).

Antibacterial Activity							
		*B.c.*	*S.a.*	*L.m.*	*E.c.*	*P.a.*	*S.t.*
**WO**	**MIC**	0.10	0.20	0.20	0.075	0.20	0.20
	**MBC**	0.20	0.40	0.40	0.10	0.40	0.40
**WP**	**MIC**	0.05	0.10	0.15	0.075	0.20	0.20
	**MBC**	0.10	0.20	0.20	0.10	0.40	0.40
**Antifungal Activity**							
		***A.fun.***	***A.v.***	***A.n.***	***P.f.***	***P.o***	***P.v.c***
**WO**	**MIC**	0.025	0.025	0.05	0.025	0.025	0.025
	**MFC**	0.05	0.05	0.10	0.05	0.020	0.050
**WP**	**MIC**	0.025	0.025	0.025	0.025	0.025	0.05
	**MFC**	0.05	0.05	0.05	0.05	0.05	0.10

B.c.: *Bacillus cereus*; S.a.: *Staphylococcus aureus*; L.m.: *Listeria monocytogenes*; E.c.: *Escherichia coli*; P.a.: *Pseudomonas aeruginosa*; S.t.: *Salmonella typhimirium*; A.fum.: *Aspergillus fumigatus*; A.v.: *Aspergillus versicolor*; A.n.: *Aspergillus niger*; P.f.: *Penicillium funiculosum*; P.o.: *Penicillium ochrochloron*; P.v.c.: *Penicillium verrucosum var. cyclopium*.

## Supplementary Materials

Figure S1: Exemplificative chromatographic profiles of *Impatiens walleriana* samples orange colour_WO (A) and pink colour_WP (B), recorded at 280 nm (A1 and B1, respectively) and 520 nm (A2 and B2, respectively), Figure S2: TIC spectrum and UV-vis spectra of the major non-anthocyanin phenolic found in both samples (p-coumaric acid hexoside *m*/*z* 325, peak 3, A) and the major anthocyanin compounds found in Impatiens walleriana samples orange colour_WO (pelargonidin-*O*-*p*-coumaroyl-hexoside-*O*-acetyl-hexoside, *m*/*z* 783, B) and pink colour_WP (malvidin-3-*O*-*p*-coumaroylhexoside-*O*-hexoside, *m*/*z* 801, C).

## Sample Availability

Not available.
