# Supplementary material for: Chemical Composition and Bioactive Characterisation of Impatiens walleriana
Source: Molecules. 2021 Mar 3;26(5):1347. doi: 10.3390/molecules26051347 (PMC7962038; doi:10.3390/molecules26051347)
Supplement: Supplementary file 1 [file molecules-26-01347-s001.pdf]

Supplementary Material.

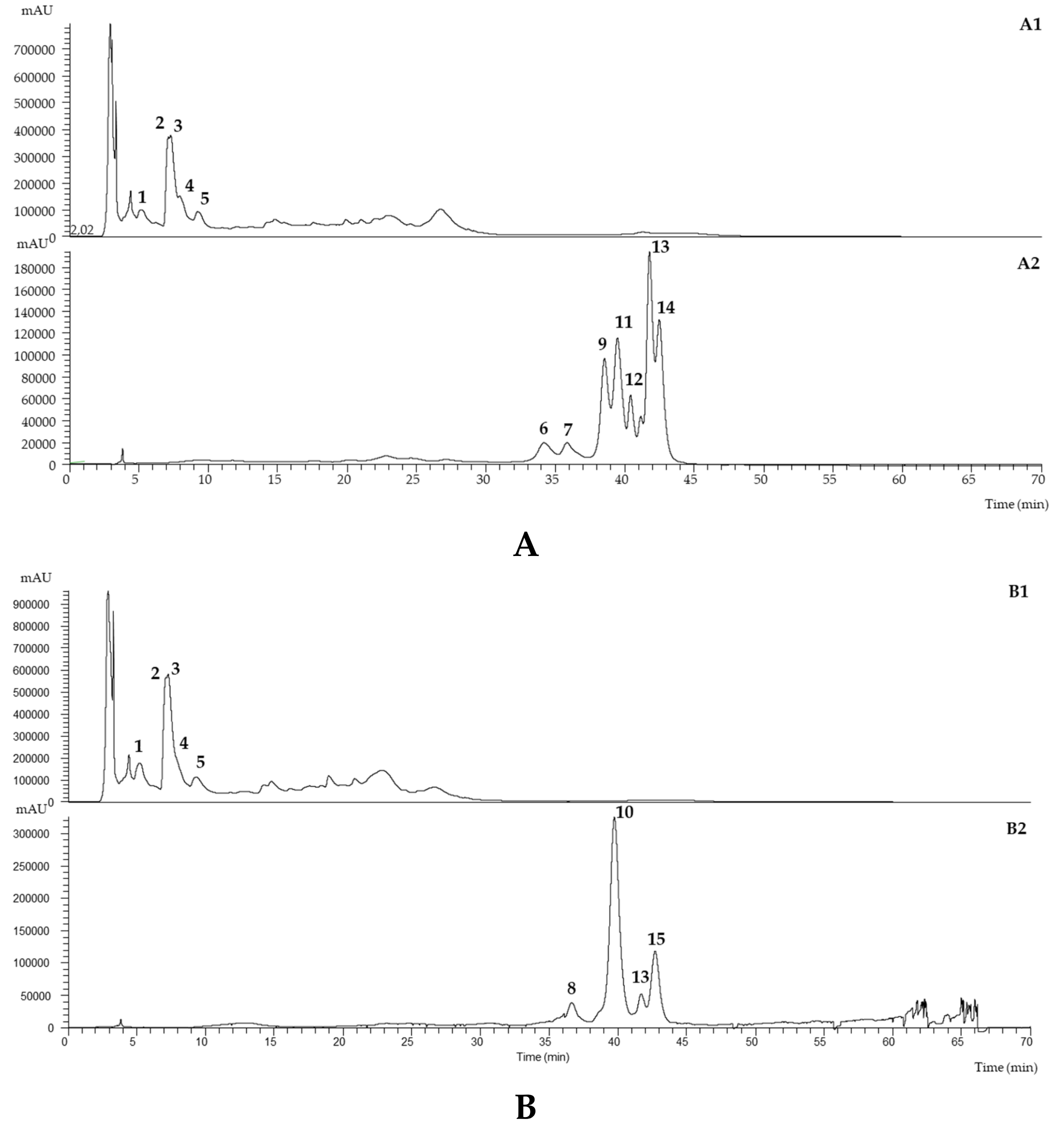

Figure S1. Exemplificative chromatographic profiles of *Impatiens walleriana* samples orange colour_WO (**A**) and pink colour_WP (**B**), recorded at 280 nm (**A1** and **B1**, respectively) and 520 nm (**A2** and **B2**, respectively).

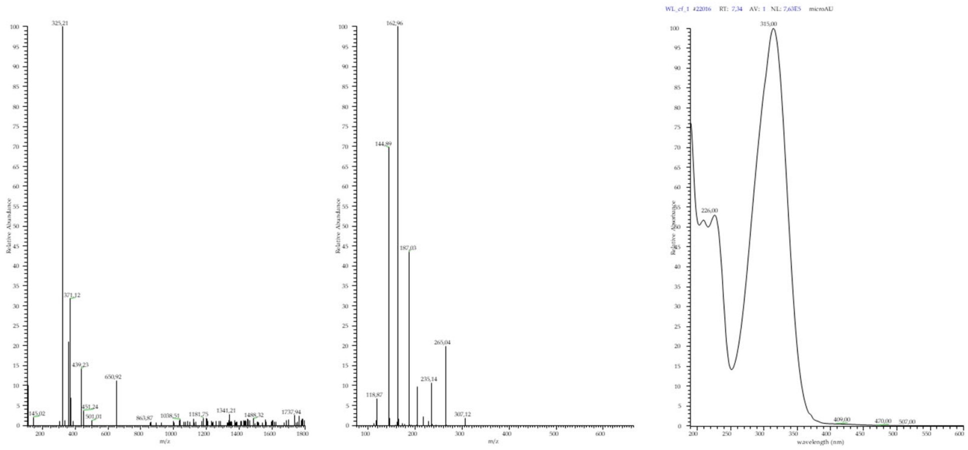

A

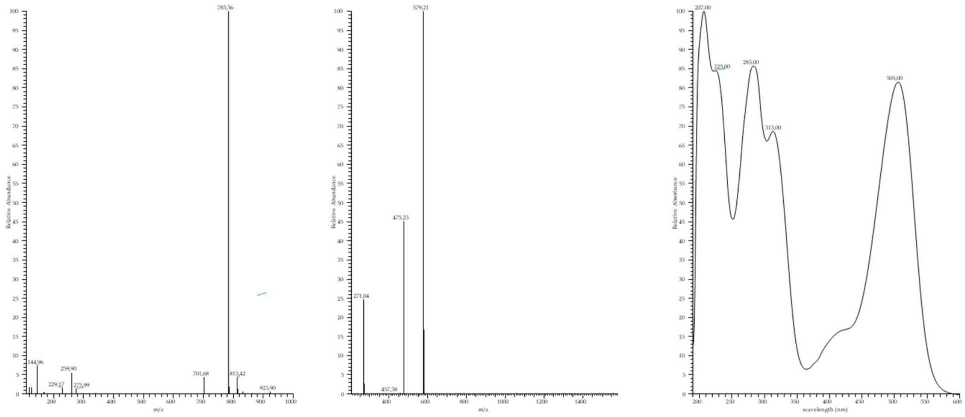

B

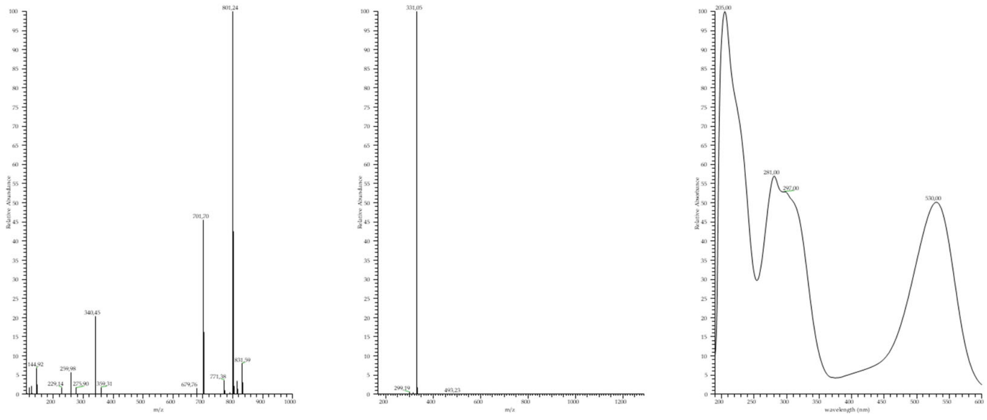

C

Figure S2. TIC spectrum and UV-vis spectra of the major non-anthocyanin phenolic found in both samples ((*p*-coumaric acid hexoside *m/z* 325, peak 3, **A**) and the major anthocyanin compounds found in *Impatiens walleriana* samples orange colour_WO (pelargonidin-*O*-*p*-coumaroyl-hexoside-*O*-acetyl-hexoside, *m/z* 783, **B**) and pink colour_WP (malvidin-3-*O*-*p*-coumaroylhexoside-*O*-hexoside, *m/z* 801, **C**).
